# UCH-L1-mediated Down-regulation of Estrogen Receptor α Contributes to Insensitivity to Endocrine Therapy for Breast Cancer

**DOI:** 10.7150/thno.39814

**Published:** 2020-01-01

**Authors:** Xi-Sha Chen, Kuan-Song Wang, Wei Guo, Lan-Ya Li, Pian Yu, Xin-Yuan Sun, Hai-Yan Wang, Yi-Di Guan, Yong-Guang Tao, Bo-Ni Ding, Ming-Zhu Yin, Xing-Cong Ren, Yi Zhang, Ce-Shi Chen, Yuan-Chao Ye, Jin-Ming Yang, Yan Cheng

**Affiliations:** 1 Xiangya School of Pharmaceutical Sciences, Central South University, Changsha, 410013, China; 2 Department of Pharmacy, the Second Xiangya Hospotal, Central South University, Changsha, 410011, China; 3 Department of Pathology, Xiangya hospital and Department of Pathology, School of Basic Medicine, Central South University, Changsha, 410078, China; 4 Cancer Research Institute, School of Basic Medicine, and Key Laboratory of Carcinogenesis and Cancer Invasion, Ministry of Education, Central South University, Changsha, 410078, China; 5 Department of Breast and Thyroid, The Third Xiangya Hospital, Central South University, Changsha, 410078, China; 6 Department of Dermatology, Hunan Engineering Research Center of Skin Health and Disease, Hunan Key Laboratory of Skin Cancer and Psoriasis, Xiangya Hospital, Central South University, Changsha, Hunan 410008, China; 7 Department of Cancer Biology and Toxicology, College of Medicine, Markey Cancer Center, University of Kentucky, Lexington, KY 40536, USA; 8 Department of Pharmacology, College of Pharmaceutical Sciences, Soochow University, Suzhou, China, 215000, China; 9 Key Laboratory of Animal Models and Human Disease Mechanisms of Chinese Academy of Sciences and Yunnan Province, Kunming Institute of Zoology, Chinese Academy of Sciences, Collaborative Innovation Center for Cancer Medicine, Kunming, 650223, China; 10 Department of Internal Medicine, University of Iowa Carver College of Medicine, Iowa City, IA, 52242,USA.; Xi-Sha Chen and Kuan-Song Wang contributed equally to this work.

**Keywords:** Ubiquitin carboxyl terminal hydrolase-L1, Estrogen receptor α, EGFR, ER-negative breast cancer, Endocrine therapy

## Abstract

**Purpose**: To determine the role of UCH-L1 in regulating ERα expression, and to evaluate whether therapeutic targeting of UCH-L1 can enhance the efficacy of anti-estrogen therapy against breast cancer with loss or reduction of ERα.

**Methods**: Expressions of UCH-L1 and ERα were examined in breast cancer cells and patient specimens. The associations between UCH-L1 and ERα, therapeutic response and prognosis in breast cancer patients were analyzed using multiple databases. The molecular pathways by which UCH-L1 regulates ERα were analyzed using immunoblotting, qRT-PCR, immunoprecipitation, ubiquitination, luciferase and ChIP assays. The effects of UCH-L1 inhibition on the efficacy of tamoxifen in ERα (-) breast cancer cells were tested both* in vivo* and *in vitro*.

**Results**: UCH-L1 expression was conversely correlated with ERα status in breast cancer, and the negative regulatory effect of UCH-L1 on ERα was mediated by the deubiquitinase-mediated stability of EGFR, which suppresses ERα transcription. High expression of UCH-L1 was associated with poor therapeutic response and prognosis in patients with breast cancer. Up-regulation of ERα caused by UCH-L1 inhibition could significantly enhance the efficacy of tamoxifen and fulvestrant in ERα (-) breast cancer both *in vivo* and *in vitro*.

**Conclusions**: Our results reveal an important role of UCH-L1 in modulating ERα status and demonstrate the involvement of UCH-L1-EGFR signaling pathway, suggesting that UCH-L1 may serve as a novel adjuvant target for treatment of hormone therapy-insensitive breast cancers. Targeting UCH-L1 to sensitize ER negative breast cancer to anti-estrogen therapy might represent a new therapeutic strategy that warrants further exploration.

## Introduction

Breast cancer is the most common malignancy and remains the leading cause of cancer death among women worldwide 1. Estrogen, a steroid hormone, has a crucial role in proliferation and growth of the mammary epithelial cells and breast cancer cells through regulating the cell cycle-related genes expression 2, 3. Estrogen-stimulated cell growth is mediated by binding to estrogen receptor (ER), ERα and ERβ, two isoforms of ER and members of the nuclear receptor superfamily of ligand-dependent transcription factors 4-7. In particular, ERα is the dominant form expressed in breast and plays an important role in the occurrence, pathological development and treatment of breast cancers, thus is considered as one of the ideal targets for treatment of breast cancers 8-13.

Breast cancers present as ERα positive (+) or negative (-) or vary in the level of ERα. ERα (+) breast cancers usually have a better prognosis and respond to endocrine therapy such as tamoxifen, a commonly used anti-estrogen agent in the treatment of breast cancer 14. However, one third of metastatic breast cancers initially responds to anti-estrogen therapy but subsequently loses ERα expression and acquires resistance to hormonal therapy 15, 16. ERα (-) breast cancers also have worse clinical outcome than ERα (+) breast cancers. Since ERα expression is essential for anti-estrogen therapy, it will be of great importance to investigate the mechanisms of ERα loss or reduction in breast cancer cells and explore effective interventions to restore ERα expression in those malignancies. Several lines of evidence indicate that restoration of ERα expression is able to induce anti-estrogen response in ERα (-) breast cancer cells 17. For example, inhibition of hyperactive MAPK by U0126, a MAPK/ERK kinase1/2 inhibitor, resulted in restoration of ERα mRNA and protein in ERα (-) breast cancer cells and conferred their sensitivity to the antiestrogen agents tamoxifen and faslodex 18, 19. Depletion of AKT3 induced ERα re-expression and increased the effectiveness of tamoxifen in ErbB2^+^/ ERα^-^ breast cancer cells 20. Genistein, a natural soybean isoflavone compound, has been shown to reactivate ERα in ERα (-) breast cancer cells and enhanced the anti-cancer efficacy of tamoxifen *in vivo* and *in vitro* 21. Recently, it was reported that genetic or pharmacological targeting of PDFG-CC sensitizes the ERα (-) breast tumors to hormone therapy through conversing basal-like breast cancers into a hormone receptor-positive state 22. Thus, a better understanding of the molecular mechanisms involved in regulation of ERα could help develop more effective therapeutic strategies to treatment of breast cancer with ERα loss or reduction.

Ubiquitin carboxyl terminal hydrolase-L1 (UCH-L1), also known as protein gene product 9.5 (PGP9.5), is a member of ubiquitin c-terminal hydrolases family. UCH-L1 belongs to deubiquitinases and is responsible for hydrolyzing carboxyl terminal esters and amides of ubiquitin 23. UCH-L1 is abundant in brain, and is associated with neurodegenerative disorders such as Parkinson**^'^**s and Alzheimer**^'^**s disease 24, 25. Notably, accumulating evidence has showed that UCH-L1 is overexpressed in various tumors including leukemia, pancreatic cancer, prostate cancer, medullary thyroid carcinoma, non-small cell lung carcinoma and colorectal cancer, and is correlated with cancer cell proliferation and metastasis 26-35. Also, expression of UCH-L1 was found to be negatively correlated with the prognosis of pancreatic, colorectal and breast cancers, and mediate multi-drug resistance in breast cancer 26, 36-40. Interestingly, there is evidence that UCH-L1 mRNA level is inversely associated with ERα status and is linked to recurrence in patients with invasive breast cancer 41; nevertheless, whether UCH-L1 has a functional role in the regulation of ERα expression remains unknown. Here, we report that UCH-L1 can deubiquitinate and stabilize EGFR, which inhibits ERα expression by transcriptional repression, and that silencing UCH-L1 expression or inhibiting the enzyme activity can up-regulate ERα expression and enhance the sensitivity of ERα (-) breast cancer cells to tamoxifen and fulvestrant. This study not only uncovers UCH-L1 as a critical regulator of ERα expression and the underlying mechanism, but also provides a potential adjuvant target for anti-estrogen therapy of breast cancer.

## Materials and Methods

### Cell lines and culture

The human breast cancer cell lines, BT549 and HCC1806, were cultured in RPMI-1640 medium, MCF-7, MCF-7/AdrR, T47D and MDA-MB-436 were cultured in DMEM medium. All cell culture media were supplemented with 10% fetal bovine serum, 100 units/mL penicillin and 100 µg/mL streptomycin. All cell lines were maintained at 37^o^C in a humidified atmosphere containing 5% CO_2_/95% air. Cell lines were authenticated using STR profile analysis and used within 3 to 20 passages of thawing the original stocks.

### Reagents and antibodies

Tamoxifen and MG132 were purchased from Sigma. Cycloheximide (CHX) was purchased from Amresco. LDN-57444 was purchased from Calbiochem. Fulvestrant and 17β-estradiol (E2) were purchased from Selleck. Antibodies used in immunoblotting: UCH-L1 (No.13179, 1:1000), ERα (No.8644, 1:1000), EGFR (No.4267, 1:1000), HA (No.3724, 1:1000) and pTyr1068-EGFR (No.3777, 1:1000) were purchased from Cell Signaling Technologies. Anti-GST (10000-0-AP, 1:4000), anti-Flag (66008-3-lg, 1:5000), anti-Myc (16286-1-AP, 1:2000), anti-UCH-L1 (66230-1-lg, 1:2000) and anti-β-actin (60008-1-lg, 1:5000) were purchased from Proteintech. Anti-eEF2K (ab45168, 1:1000) was purchased from Abcam. Anti-pThr678-EGFR (orb14895, 1:1000) was purchased from Biorbyt. Normal IgG/Peroxidase-conjugated AffiniPure Goat Anti-Rabbit/Mouse IgG (H+L) was purchased from Jackson Immuno Research.

### siRNA, shRNA and plasmid transfection

siRNA targeting UCH-L1 was purchased from Santa Cruz Biotechnology. Transfection of siRNA was carried out according to the manufacturer's protocol. Briefly, cells in exponential phase of growth were plated in six-well tissue culture plates at 1x 10^5^ cells per well, grown for 24 h, and then transfected with siRNA using lipofectamine RNAimax reagent and OPTI-MEM I-reduced serum medium. To stably silence UCH-L1 expression, the UCH-L1-targeted shRNA lentiviral particles (GENE) were transduced into cells, and the cells stably expressing the shRNA were then selected with 1 μg/mL of puromycin for 7 days. Transfection of the plasmid was carried out using lipofectamine 2000 (Invitrogen) reagent according to the manufacturer's protocol.

### Western blot analysis

Cells were lysed at ice for 30 minutes in RIPA supplemented with a protease inhibitor cocktail (Biotool), followed by centrifugation at 12,000 x g for 15 minutes. Proteins (20-40 µg) were resolved by SDS-PAGE and then transferred to PVDF membrane. The PVDF membranes were incubated with the respective antibodies in 5% BSA at 4^o^C overnight, followed by incubation with a secondary antibody at room temperature for 1 h. The protein signals were detected by ECL method.

### Luciferase Reporter Assay

To analyze ERE promoter activity, HCC1806 cells stably expressing the indicated shRNA were co-transfected with the ERE-containing luciferase reporter plasmid or identical NON-Luc construct plus pRL-TK-Luc to assess transfection efficiency, and then treated with 10nM E2 for 24h. Cells were harvested and the luciferase activities were measured using Promega's Luciferase Assay System (Promega, Madison, Wisconsin). The luciferase values (relative light units, RLUs) were normalized based on the activity of pRL-TK-Luc.

### Chromatin Immunoprecipitation Assay

The chromatin or DNA-protein complex was isolated according to the manufacturer's instruction (Abcam, ab117152-Chromatin Extraction Kit). Then Chromatin immunoprecipitation (CHIP) experiment was carried out by using a ChIP assay kit according to the manufacturer's instructions (Abcam, ab117138-ChIP Kit-One Step). Quantitative real-time polymerase chain reaction (qPCR) was performed to measure the ChIP signal, and enrichment of target was analyzed based on input DNA and normal IgG signals. The following specific primers were used in the CHIP-qPCR analysis: NRIP1 promoter (5'- TGCTCCTGGGTCCTACGTCT-3' and 5'-TCCCCTTCACCCCACAACAC-3'), CCND1 promoter (5'- AGCTTTCCATTCAGAGGTGTGTTTC -3' and 5'- CCTTCTAGCCTGGAGACTCTTCG -3').

### Immunoprecipitation assay

Cells were lysed with RIPA lysis buffer supplemented with a protease-inhibitor cocktail (Biotool). Immunoprecipitations were performed using the indicated primary antibody and protein A/G agarose beads (from Santa Cruz) at 4 ^◦^C overnight. The immunocomplexes were then washed four times with RIPA buffer, and proteins were boiled in SDS-PAGE sample buffer for 10 min, followed by western blotting.

### Pulse-chase assay

For the EGFR half-life assay, UCH-L1 plasmid was transfected into HEK293T cells when cells reached about 60% confluence. Twenty-four hours later, the cells were treated with the protein synthesis inhibitor cycloheximide (Amresco, 10 µg ml**^-1^**) for the indicated durations before collection. The MCF-7/AdrR, HCC1806 and BT549 cell lines transfected with the indicated shRNA or siRNA were treated with the protein synthesis inhibitor cycloheximide (Amresco, 10 µg ml**^-1^**) for the indicated durations before collection.

### GST pulldown assay

Purified GST or GST-UCH-L1 from bacteria bound to glutathione-sepharose 4B beads (from sigma) was incubated with cell lysates for 4 hours at 4 ^◦^C. The beads were then washed with GST binding buffer four times, and the bound proteins were separated by SDS-PAGE and immunoblotted with indicated antibodies.

### *In vivo* EGFR deubiquitination assay

Flag-EGFR, HA-ubiquitin and UCH-L1 plasmids or UCH-L1 siRNA were transfected into HEK293T cells with lipofectamine reagent. Transfected HEK293T cells were treated with 20μM of the proteasome inhibitor MG132 for 8 hours before being harvested. The cells were washed with PBS and lysed. The lysates were immunoprecipitated with anti-Flag antibody and protein A/G agarose beads (Santa Cruz) at 4 ^◦^C overnight. Then the beads were washed four times with RIPA buffer. The proteins were released from the beads by boiling in SDS-PAGE loading buffer and analysed by immunoblotting with anti-HA antibody.

### Deubiquitination of EGFR *in vitro*

For preparation of ubiquitinated EGFR as the substrate for the in vitro deubiquitination assay, HEK293T cells were transfected with HA-Ubiquitin, Flag-EGFR. At 2 days after transfection, the cells were treated with 20 μM MG132 for 8h to enrich the ubiquitinated EGFR proteins. Then, Flag-tagged ubiquitinated EGFR proteins were purified by immunoprecipitation and eluted with Flag peptides (Sigma). Next, the ubiquitinated EGFR proteins were incubated with purified GST or GST-UCH-L1 in a deubiquitination buffer (50mM Tris-HCl pH 8.0, 50mM NaCl, 1mM EDTA, 10mM DTT, 5% glycerol) for 4 hours at 37 ^◦^C. Ubiquitinated EGFR was detected by WB using the anti-HA antibody.

### Clonogenic assay

Cells were plated in 6-well tissue culture plates (500 cells per well) and incubated at 37°C in a humidified atmosphere containing 5% CO_2_/95% air for 15 days. At the end of incubation, cells were fixed with 4% paraformaldehyde and stained with crystal violet for 20 min, washed with PBS, and then the colonies were counted.

### 5-Ethynyl-2'- deoxyuridine assay

The cells were incubated with 5-Ethynyl-2'- deoxyuridine assay (EdU; Ribobio) for 2 hours, and processed according to the manufacturer's instruction. After three washes with PBS, the cells were incubated with 100μL of 1X Apollo reaction cocktail for 30 minutes. Then cells were washed three times with 0.5% Triton X-100. The DNA contents were stained with 100μL of 1X Hoechst 33342 (5 μg/mL) for 30 minutes and visualized under a fluorescence microscope.

### Acquisition and analysis of GEO data

Gene expression from GSE7390, GSE30682 were extracted from the NCBI Gene Expression Omnibus (GEO) database and analyzed using the R2: Genomics Analysis and Visualization Platform (http://r2.amc.nl). Normalized log2 transformed gene expression data were downloaded from the R2 platform to a Microsoft Excel spreadsheet for additional analysis.

### Immunohistochemistry staining of clinical samples

From January 2010 to July 2012, the tissues of breast cancer patients with different molecular type (Luminal A, *n*=45; Luminal B, *n*=46; Triple negative, n=29; HER2+, n=45) were collected, respectively. The practical classification of intrinsic subtypes was proposed at the St. Gallen consensus meeting of breast cancer 42, 43. All subjects enrolled in this study at the XiangYa Hospital Central South University (Changsha, China) gave written informed consent. The Institute Ethical Committee approved the study protocol according to the guidelines of Helsinki conventions.

IHC staining for UCHL1, ERα, PR and HER2 was performed on formalin-fixed paraffin-embedded tissues using the DAKO LSAB+System-HRP kit (DAKO), according to the manufacturer's instructions. UCHL1 was detected with a commercially available rabbit polyclonal antibody at a dilution of 1:200. To evaluate ERα expression, we used rabbit polyclonal antibody SP1 (dilution 1:200, Abcam). Anti-PR antibody SP2 (dilution 1:150, Abcam) was used for IHC of PR. Anti-HER2 antibody SP3 (dilution 1:200, Abcam) was used for IHC of HER2. IHC staining was assessed by two independent pathologists under blinded conditions. The intensity of UCHL1 staining was evaluated using the following criteria: we regarded ≥10% of cells exhibiting cytoplasm staining as positive expression (+); <10% cytoplasm staining of tumor cells was considered as negative expression (-).

### Tamoxifen sensitivity analysis

Data transformed to Z-score of Tamoxifen and UCHL1 mRNA expression was downloaded from the Cell Miner Database Version: 2.1 (http://discover.nci.nih.gov/cellminer/). NSC identifiers were 180973 for Tamoxifen. Correlation between mRNA expressions of 59 cancer cell lines with drug sensitivity in them was done with a regression analysis and correlation coefficient was estimated.

### The kaplan-meier plotter

The prognostic value of UCHL1 mRNA expression was evaluated using an online database, Kaplan-Meier Plotter (www.kmplot.com) 44 which contained gene expression data and survival information of clinical breast cancer patients who received TAM as their only endocrine therapy. To analyze the distant metastasis-free survival (DMFS) of breast cancer patients treated with tamoxifen, patient samples were split into two groups by median expression (high vs. low expression) and assessed by a Kaplan-Meier survival plot, with the hazard ratio (HR) with 95% confidence intervals (CI) and log rank p value.

### Quantitative real-time PCR

Total RNAs were isolated from cells using the Trizol reagent (Biotech) and 1st strand cDNA was synthesized using PrimeScript RT Reagent Kit (Perfect real time) (Takara). Real time PCR was performed using SYBR Premix Ex Tap (Tli RNaseH Plus) (Takara), and was run on Bio-Rad. For quantification of gene expression, the 2**^-ΔΔCt^** method was used. GAPDH expression was used for normalization. The qPCR primer sets: ERα: 5'-CCTGATGATTGGTCTCGTCTG-3' (forward) and 5'-GGCACACAAACTCCTCTCC-3' (reverse); CCND1: 5'-TGCATCTACACCGACAACTCC-3' (forward) and 5'-CGTGTTTGCGGATGATCTGTT-3' (reverse); AGR2: 5'-ATGGAGAAAATTCCAGTGTC-3' (forward) and 5'-TTACAATTCAGTCTTCAGCA-3' (reverse); GAPDH: 5'-ACCACAGTCCATGCCATCAC-3' (forward) and 5'-TCCACCACCCTGTTGCTGTA-3' (reverse).

### Cellular viability assay

Cells were plated at 5x10^3^ cells per well in 96-well tissue culture plates, subjected to different treatments, and then incubated at 37^o^C for indicated time in a humidified atmosphere containing 5% CO_2_/95% air. Cell viability was measured using CCK-8 assay.

### Statistical analysis

The difference between the samples with or without silencing of UCH-L1 expression was analyzed using unpaired two-tailed Student's *t*-test. All experiments were performed at least three times. *P* values <0.05 were considered statistically significant.

### Study approval

Animal studies were approved by the Ethics Committee of Xiangya School of Pharmaceutical Sciences, and the animal protocol was in accordance with the institutional guidelines of the Animal Care and Use Committee of Central South University. Briefly, the HCC1806 breast cancer cells were injected subcutaneously into female nude mice (2×10^6^ cells in 100 μl per inoculation). Tumor volume was calculated as length × width^2^× (π/ 6). When the tumors were palpable, mice were alternately divided into four groups (n=6/group). When the mean diameter of tumors reached 5-6 mm, the mice received indicated treatment. Tumor sizes and body weights were measured every other day.

## Results

### UCH-L1 expression conversely correlates with ERα status in breast cancers

**Figure 1:**
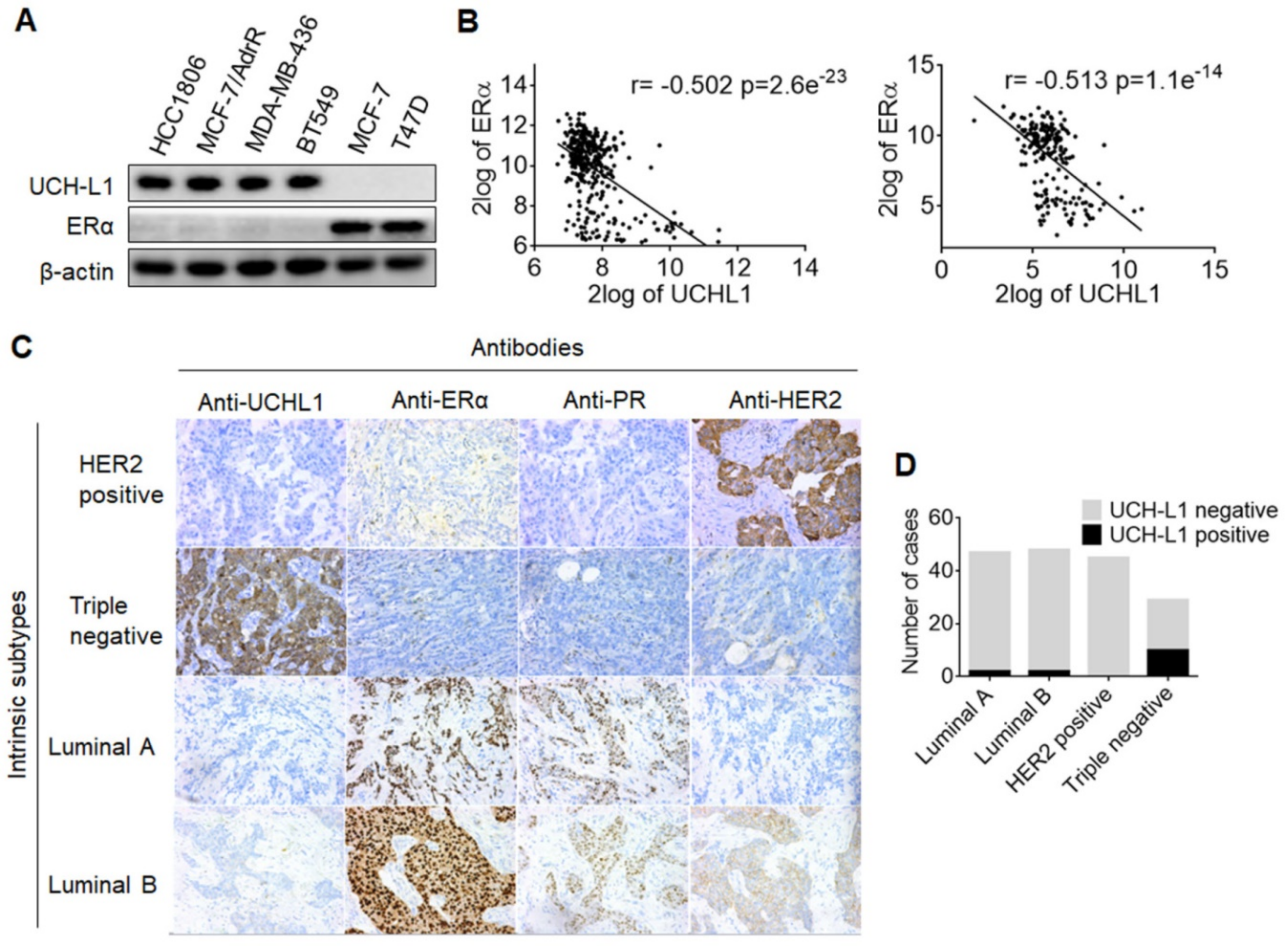
** The converse correlation between UCH-L1 and ERα. (A)** The expressions of UCH-L1 and ERα in ERα (-) and ERα (+) breast cancer cells were measured by western blot. β-actin was used as a loading control. **(B)** Correlation between UCHL1 and ERα mRNA levels in GSE30682 (left) and GSE7390 (right) breast cancer samples.** (C)** A total of 169 clinical human breast carcinoma cases were subjected to immunohistochemical analyses with UCH-L1 antibody. The UCH-L1 expressions in representative tumor tissues including luminal A, luminal B, triple negative, and HER2 overexpression.** (D)** Immunohistochemical analyses of UCH-L1 expression in patients specimens.

In a proteomic comparison of ERα (+) MCF-7 and ERα (-) MCF-7/AdrR cells, we found that UCH-L1 was abundant in MCF-7/AdrR cells, but not detectable in MCF-7 cells (Figure S1). These observations prompted us to explore whether there is a relationship between expressions of UCH-L1 and ERα. We first measured and compared the expressions of UCH-L1 in six human breast cancer cell lines. As shown in Figure 1A, UCH-L1 was abundantly expressed in the ERα (-) cell lines HCC1806, MCF-7/AdrR, MDA-MB-436 and BT549; by contrast, this deubiquitinating enzyme was barely detectable in the ERα (+) cell lines, MCF-7 and T47D. We then conducted a search and analysis of two data sets of breast cancer mRNA expression, GSE30682 45 and GSE7390 46, on the GEO using the online tool R2: Genomics Analysis and Visualization Platform (http://r2.amc.nl/). These analyses revealed an inverse association between UCH-L1 and ERα in breast cancer (Figure 1B). To determine the clinical implication of these results, we analyzed the expressions of UCH-L1 and ERα in the specimens from breast cancer patients. We observed that the rate of positive expression (+) of UCHL1 protein is significantly higher in triple negative breast tumors (34.5%, 10/29) than that in luminal A (4.3%, 2/47), luminal B (4.2%, 2/48) and HER2+ (0%, 0/45) breast tumors. Notably, HER2+ breast cancer has low expressions of both ERα and UCH-L1 (Figure 1C-D; Table S1). These data suggest that loss or reduction of ERα in breast cancer may be causally associated with the up-regulation of UCH-L1.

### UCH-L1 negatively affects ERα expression in breast cancer cells

**Figure 2:**
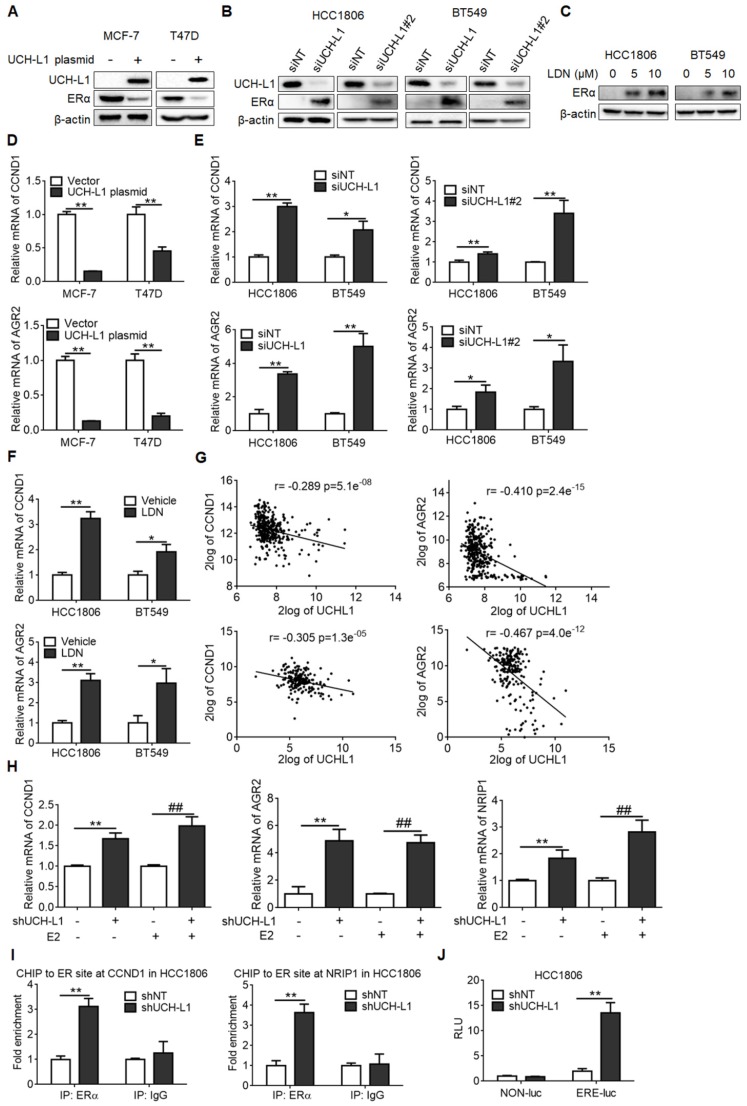
** UCH-L1 negatively regulates ERα in breast cancer cells. (A)** MCF-7 or T47D cells were transfected with a control plasmid or a myc-his-UCH-L1 plasmid for 48h. **(B and C)** HCC1806 or BT549 cells were transfected with a non-targeting siRNA or UCH-L1 siRNAs for 72h **(B)**, or were treated with UCH-L1 inhibitor LDN with the indicated concentrations for 24h **(C)**. The expressions of UCH-L1 and ERα were measured by western blot. β-actin was used as a loading control.** (D)** MCF-7 or T47D cells were transfected with a control plasmid or a myc-his-UCH-L1 plasmid for 48h. **(E and F)** HCC1806 or BT549 cells were transfected with a non-targeting siRNA or UCH-L1 siRNAs for 72h **(E)**, or were treated with 10 μM LDN for 24h** (F)**. The mRNA levels of CCND1 and AGR2 were analyzed by real-time PCR (Mean ± s.d., n=3 biologically independent experiments. ∗, *p <*0.05; ∗∗, *p <*0.01). **(G)** Correlation between UCHL1 and AGR2, CCND1 mRNA levels in GSE30682 (upper) and GSE7390 (bottom) breast cancer samples.** (H)** The mRNA levels of ER-target genes in control or UCH-L1 knockdown HCC1806 cells following treatment with vehicle or 10nM E2 for 24 hours, were analyzed by real-time PCR (Mean ± s.d., n=3. ∗∗, *p <*0.01; ##, *p <*0.01 compared with E2). **(I)** ChIP-qPCR analysis. Fold enrichment of ERα at the CCND1/NRIP1 promoter regions in the presence of 10nM E2 for 24 hours (Mean ± s.d. of triplicate measurements. ∗∗, *p <*0.01). **(J)** ERE-luciferase assay in the control or UCH-L1 knockdown HCC1806 cells in the presence of 10nM E2 for 24 hours (Mean ± s.d., n=3. ∗∗, *p <*0.01).

To determine if expression of UCH-L1 indeed affects ERα, we overexpressed UCH-L1 using an UCH-L1 expression plasmid or knocked down UCH-L1 using RNA interference, and then compared the content of ERα in the breast cancer cells with different levels of UCH-L1. As shown in Figure 2A, transfection of the ERα (+) breast cancer cells with an UCH-L1 expression plasmid resulted in a remarkable reduction of ERα amount. Conversely, knockdown of UCH-L1 expression using a siRNA or treatment of cells with a specific inhibitor of UCH-L1, LDN-57444 (LDN), caused a significant increase in ERα expression (Figure 2B-C). Similar results were obtained in MCF-7/AdrR and MDA-MB-436 cells (Figure S2A-B). These data suggest that UCH-L1 has an inhibitory effect on the expression of ERα. To verify the effect of UCH-L1 on ERα expression, we measured the mRNA levels of CCND1 and AGR2, two target genes of ERα, following manipulating UCH-L1 expression. Figure 2D shows that overexpression of UCH-L1 in the ERα (+) breast cancer cells (MCF-7 and T47D) dramatically reduced the mRNA levels of CCND1 and AGR2; in contrast, knockdown of UCH-L1 in the ERα (-) breast cancer cells increased the mRNA levels of these two genes (Figure 2E; Figure S2C). Elevation of CCND1 and AGR2 mRNA levels were also observed in the ERα (-) breast cancer cells treated with LDN (Figure 2F; Figure S2D). Analyses of GSE30682 and GSE7390 data sets revealed an inverse relationship between expressions of UCH-L1 and AGR2/CCND1 (Figure 2G). These results further support the negative regulation of ERα expression by UCH-L1 in breast cancer cells.

Upon binding to its ligand, ERα dimerizes, localizes to the nuclear and binds to the estrogen response elements (ERE) in the promoter regions of estrogen regulated genes, thus enhancing the transcription of target genes. We next asked whether ERα re-expression in ER-negative cells upon UCH-L1 inhibition would elicit classical ER-like transactivation activity upon estrogen treatment. Figure 2H shows that knockdown of UCH-L1 upregulated the mRNA levels of ER-responsive genes in the cells treated with estrogen. Chromatin immunoprecipitation (CHIP) assays revealed that inhibition of UCH-L1 increased the recruitment of ERα to the promoter regions of NRIP1 and CCND1 in the ER-negative cells upon estrogen treatment (Figure 2I; Figure S2E). In addition, ERE luciferase assay showed that inhibition of UCH-L1 enhanced ERα transcriptional activity in the ER-negative cells treated with estrogen (Figure 2J). These results indicate that ERα restoration by UCH-L1 repression is functionally active.

### Regulation of ERα by UCH-L1 is mediated via EGFR pathway

**Figure 3:**
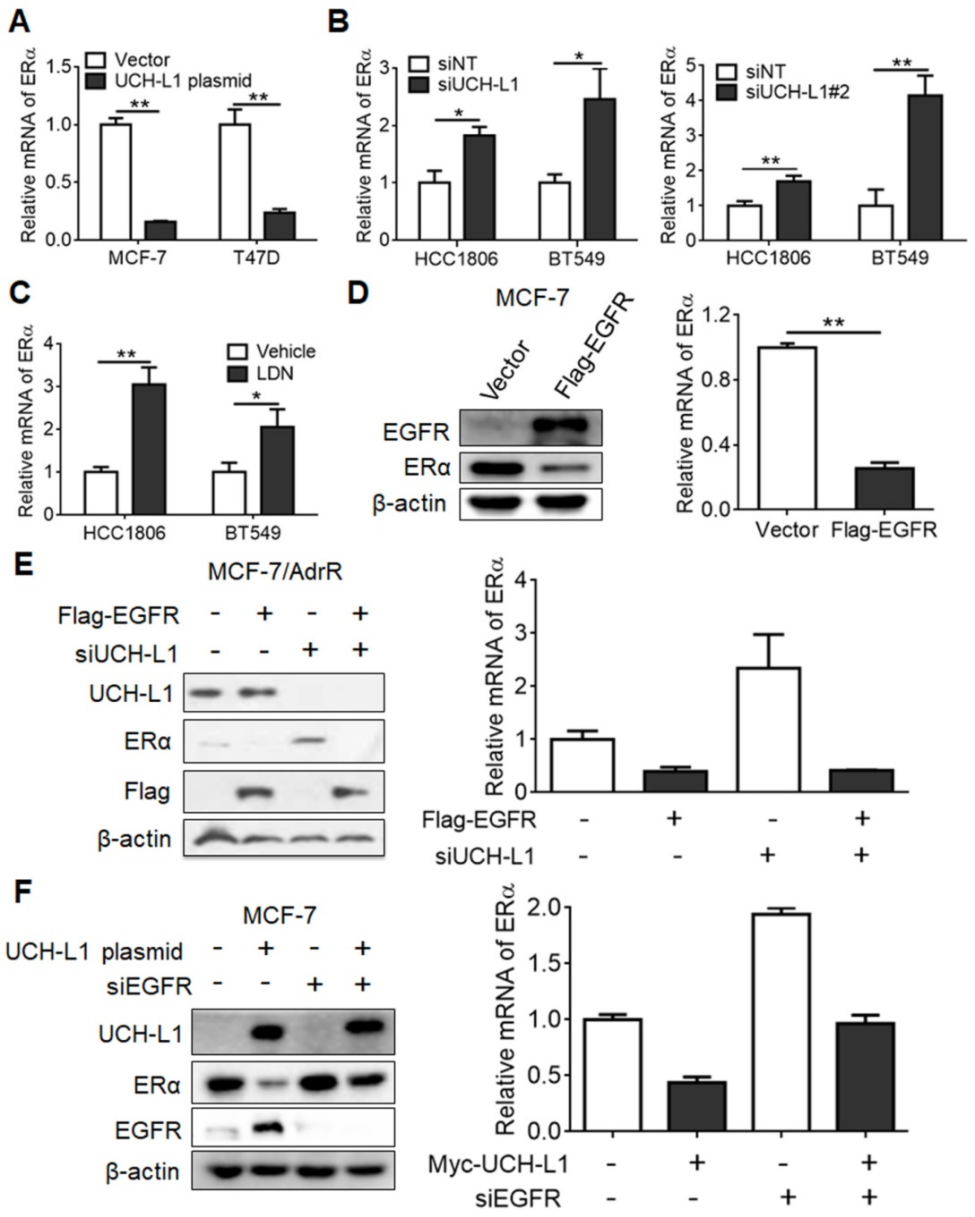
** UCH-L1 regulates the transcription of ERα gene via EGFR pathway. (A)** MCF-7 or T47D cells were transfected with a control plasmid or a myc-his-UCH-L1 plasmid. **(B and C)** HCC1806 or BT549 cells were transfected with a non-targeting siRNA or UCH-L1 siRNAs for 72h **(B)**, or were treated with 10 μM LDN for 24h **(C)**. The ERα mRNA level was analyzed by real-time PCR.** (D)** MCF-7 cells were transfected with a control plasmid or a Flag-EGFR plasmid. The mRNA level of ERα was measured by real-time PCR. The expressions of EGFR and ERα were measured by western blot. β-actin was used as a loading control. Results shown are Mean ± s.d., n=3. ∗, *p <*0.05; ∗∗, *p <*0.01. **(E)** MCF-7/AdrR cells were transfected with a non-targeting siRNA or an UCH-L1 siRNA, followed by transfection with a Flag-EGFR expression plasmid. **(F)** MCF-7 cells overexpressing UCH-L1 were transfected with a non-targeting siRNA or an EGFR siRNA. The mRNA level of ERα was measured by real-time PCR. The expressions of UCH-L1, ERα and EGFR were measured by western blot. β-actin was used as a loading control.

We next sought to understand how UCH-L1 regulates ERα expression. Firstly, we explored the possible role of UCH-L1 in ERα degradation through use of the proteasome inhibitor MG132. MG132 could not rescue the down-regulation of ERα in MCF-7 cells transfected with an UCH-L1 expression plasmid (Figure S3), suggesting that this deubiquitinase does not affect the stability of ERα protein. We next determined the effect of UCH-L1 on ERα mRNA. Ectopic expression of UCH-L1 reduced the mRNA level of ERα (Figure 3A), but knockdown of UCH-L1 or treatment with LDN resulted in an elevated level of ERα mRNA (Figure 3B-C; Figure S4). These results suggest that UCH-L1 affects the expression of ERα at the transcription level. As EGFR was reported to suppress ERα gene transcription 18, and UCH-L1 may participate in regulating EGFR protein expression 47, we then determined the possible involvement of EGFR in the regulation of ERα expression by UCH-L1. We found that the amounts of both protein and mRNA of ERα were decreased in MCF-7 cells subjected to transfection of an EGFR vector (Figure 3D). Moreover, the up-regulations of ERα mRNA and protein expressions in the UCH-L1 knockdown cells were abrogated by ectopic expression of EGFR (Figure 3E). Inversely, the decrease of ERα induced by UCH-L1 overexpression was reversed by EGFR silencing (Figure 3F). These results suggest an essential role for EGFR in mediating the effect of UCH-L1 on ERα expression.

### UCH-L1 deubiquitinates EGFR to promote its stability

**Figure 4:**
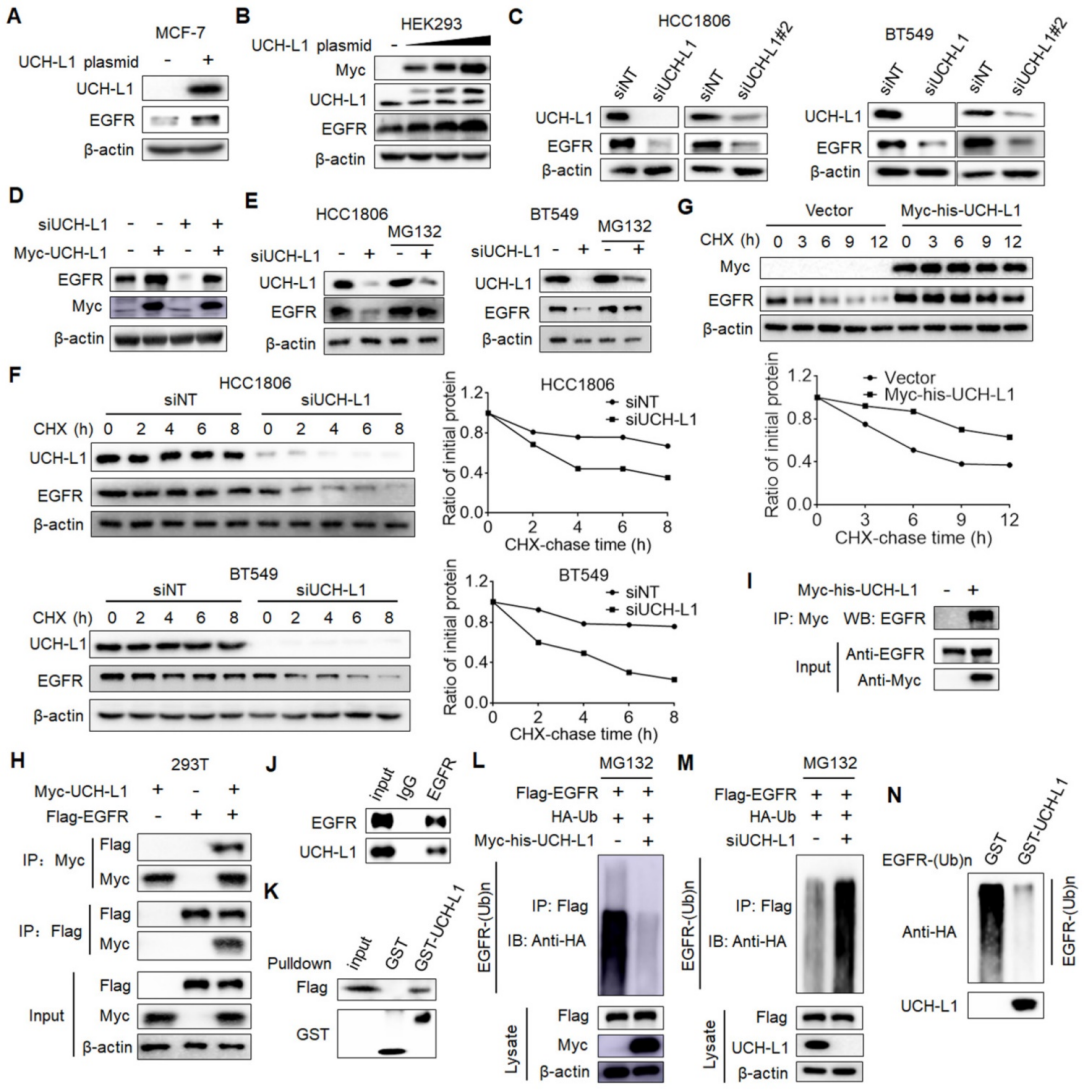
** UCH-L1 deubiquitinates and stabilizes EGFR. (A)** MCF-7 cells were transfected with a control plasmid or a myc-his-UCH-L1 plasmid. The expressions of UCH-L1 and EGFR were measured by western blot. β-actin was used as a loading control. **(B)** Increasing amounts (0μg, 0.5μg, 1.5μg, 3μg) of UCH-L1 plasmid were transfected into HEK293 cells, and the expression of EGFR was measured by western blot. **(C)** HCC1806 or BT549 cells were transfected with a non-targeting siRNA or UCH-L1 siRNAs. The expressions of UCH-L1 and EGFR were measured by western blot. β-actin was used as a loading control. **(D)** MCF-7/AdrR cells were transfected with a non-targeting siRNA or an UCH-L1 siRNA, followed by transfection with a siRNA-resistant myc-his-UCH-L1 expression plasmid. The expressions of EGFR and Myc were measured by western blot. β-actin was used as a loading control. **(E)** HCC1806 or BT549 cells were transfected with a non-targeting siRNA or an UCH-L1 siRNA, followed by treatment with 20μM MG132 for 4h. The expressions of UCH-L1 and EGFR were measured by western blot. β-actin was used as a loading control. **(F and G)** HCC1806 or BT549 cells were transfected with a non-targeting siRNA or an UCH-L1 siRNA, and then subjected to cycloheximide (10μg/ml) chase at the indicated time **(F)**. HEK293T cells were transfected with a control plasmid or a myc-his-UCH-L1 plasmid, and then subjected to cycloheximide (10μg/ml) chase at the indicated time **(G)**. The expression of EGFR was measured by western blot. β-actin was used as a loading control. **(H)** HEK293T cells were transfected with Flag-EGFR and myc-his-UCH-L1 plasmids, and then subjected to immunoprecipitation with anti-Flag or anti-Myc antibodies. The lysates and immunoprecipitates were then blotted. **(I)** MCF-7/AdrR cells transfected with myc-his-UCH-L1 plasmid were subjected immunoprecipitation with anti-Myc antibodies. The lysates and immunoprecipitates were analyzed. **(J)** Endogenous UCH-L1 and EGFR proteins interact with one another in MCF-7/AdrR cells. Endogenous EGFR proteins were immunoprecipitated with the anti-EGFR antibody. Endogenous UCH-L1 was detected by WB. **(K)** HEK293T cells transfected with Flag-EGFR were lysed and lysates were incubated with GST or GST-UCH-L1-GSH-Sepharose. Proteins retained on Sepharose were blotted with the indicated antibodies. **(L and M)** HEK293T cells transfected with the indicated constructs were treated with MG132 (20μM) for 8 hours before harvest. EGFR was immunoprecipitated with anti-Flag antibodies and immunoblotted with anti-HA antibodies. **(N)** Ubiquitinated EGFR was purified from MG132-treated HEK293T cells and then incubated with purified GST or GST-UCH-L1 in vitro, and then blotted with anti-HA antibodies.

Next, we wanted to determine how UCH-L1 affects EGFR. We first validated the effects of UCH-L1 on EGFR. As shown in Figure 4A, overexpression of UCH-L1 in MCF-7 cells resulted in a significant increase in EGFR expression, and ectopic expression of UCH-L1 resulted in EGFR elevation in a dose-dependent manner (Figure 4B). Depletion of UCH-L1 by siRNA markedly decreased the protein level of EGFR (Figure 4C; Figure S5A). When we re-expressed UCH-L1 in the UCH-L1 knockdown cells, the down-regulation of EGFR in the cells subjected to silencing of UCH-L1 expression was blocked (Figure 4D). We further observed that silencing UCH-L1 expression also reduced phospho-EGFR at Thr678 and Tyr1068 (Figure S5B). Because UCH-L1 is a deubiquitinating enzyme and can affect protein stability, we tested the effect of UCH-L1 on stability of EGFR. We found that MG132, a proteasome inhibitor, could rescue the down-regulation of EGFR in the cells with knockdown of UCH-L1 expression (Figure 4E; Figure S5C). We also compared the half-life of EGFR protein in the cells with or without knockdown of UCH-L1, using the cycloheximide (CHX) chase assay. Knockdown of UCH-L1 expression decreased the half-life of EGFR protein (Figure 4F; Figure S5D), and the EGFR protein half-life was extended in cells overexpressed UCH-L1 (Figure 4G). These results demonstrate a role for UCH-L1 in stabilizing EGFR protein. The stabilizing effect of UCH-L1 on EGFR appears to be specific, as the expression of elongation factor-2 kinase (eEF2K) remained unchanged in the cells subjected to UCH-L1 silencing (Figure S6A), and knockdown of UCH-L1 did not affect the half-life of eEF2K (Figure S6B). These experiments suggest a selective role of UCH-L1 in stabilizing EGFR protein.

To analyze whether UCH-L1 stabilizes EGFR through deubiquitination of the protein, we examined the physical interaction between UCH-L1 and EGFR proteins. 293T cells transfected with a Myc-UCH-L1 vector or Flag-EGFR vector alone, or together, were subjected to immunoprecipitation with an anti-Flag or an anti-Myc antibody. Figure 4H shows that Flag-EGFR was detected in anti-Myc co-IPs from the cells co-transfected with Flag-EGFR and Myc-UCH-L1, but not in the cells expressing Flag-EGFR and Myc-vector. Additionally, Myc-UCH-L1 was presented in anti-Flag co-IPs from cells co-transfected with Myc-UCH-L1 and Flag-EGFR, but not in the cells transfected with Myc-UCH-L1 and Flag-vector (Figure 4H). We performed the similar experiments in the MCF-7/AdrR cells transfected with a Myc-his-UCH-L1 plasmid, and found that EGFR was co-immunoprecipitated with the anti-Myc antibody (Figure 4I). Further, we demonstrated the association of endogenous UCH-L1 and EGFR in MCF-7/AdrR cells (Figure 4J). The results of *in vitro* binding assays support the above observation. As shown in Figure 4K, when purified recombinant GST-UCH-L1 was pulled down by glutathione beads, Flag-EGFR was detected in the complex, suggesting a direct interaction between UCH-L1 and EGFR. We also demonstrated the deubiquitination of EGFR by UCH-L1. As shown in Figure 4L-M, ectopic expression of UCH-L1 reduced EGFR protein ubiquitination; by contrast, knockdown of UCH-L1 increased EGFR protein ubiquitination. In order to directly show the effect of UCH-L1 activity on ubiquitination of EGFR, we performed an *in vitro* deubiquitination assay. In these experiments, the ubiquitinated EGFR purified from cells expressing Flag-EGFR and HA-Ub by immunoprecipitation were incubated with purified GST or GST-UCH-L1 in a cell-free system. Figure 4N shows that the purified UCH-L1 could effectively deubiquitinate EGFR protein. These results indicate that UCH-L1promotes EGFR protein stability in a DUB activity-dependent manner.

### High UCH-L1 expression is associated with poor therapeutic response in malignancy and poor prognosis in breast cancer patients

**Figure 5:**
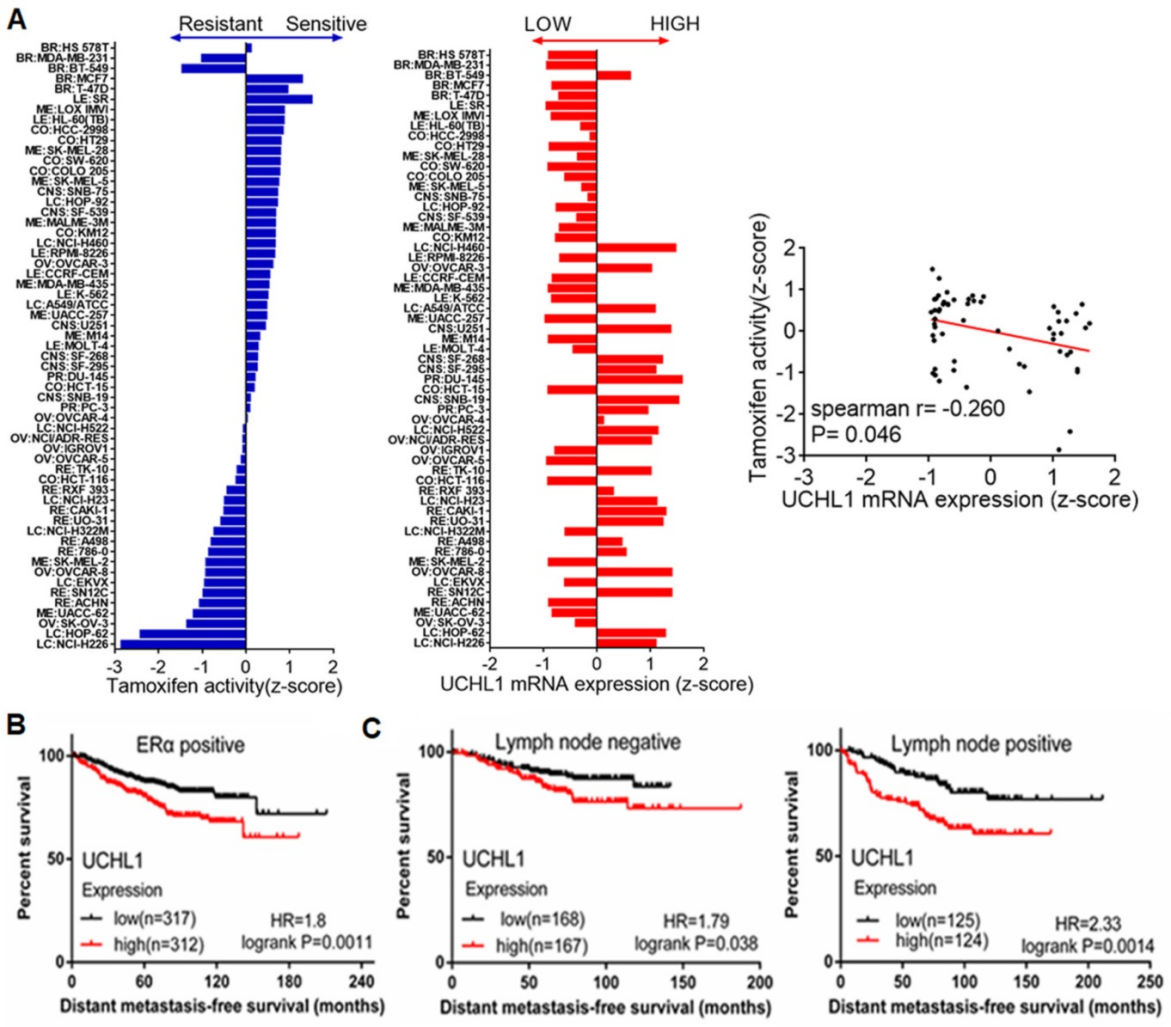
** High UCH-L1 expression is correlated with poor therapeutic outcome and prognosis in breast cancer. (A)** Evaluation of the influence of UCHL1 expression in drug activity of tamoxifen. Left: Tamoxifen drug activity in the NCI-60 cell lines. The bar graphic shows the Z-score for sensitive (0 to +3) and resistant cell lines (0 to -3). Middle: Expression of UCHL1 across the NCI-60 cell lines. The y-axis shows name of cell line and x-axis shows the expression of UCHL1. Right: The expression of UCHL1 in the 59 cancer cell lines was inversely correlated with the sensitivity to tamoxifen (spearman r=-0.260 p=0.046). **(B and C)** Determination of prognostic value of UCHL1 mRNA expression in ERα positive BC patients (DMFS in Kaplan-Meier plotter). All the patients were received TAM as their only endocrine therapy, **(B)** Kaplan-Meir survival curves for the patients with ERα positive breast cancer, **(C)** Kaplan-Meier survival curves for the patients with ER-positive and Lymph node positive or negative status.

The inhibitory effect of UCH-L1 on ERα expression implies that UCH-L1 expression may affect efficacy of anti-estrogen therapy. Indeed, Figure 5A shows that UCH-L1 expression is negatively associated with the sensitivity of cancer cells to the anti-estrogen agent tamoxifen, as evidenced by analysis of the data from the Cell Miner Analysis Tool project (http://discover.nci.nih.gov/cellminer/) in 59 cancer cell lines. We further analyzed the effect of UCH-L1 expression on the prognosis of patients treated with tamoxifen, using the online tool K-M plotter (www.kmplot.com). As shown in Figure 5B, high expression of UCH-L1 is significantly associated with poor distant metastasis-free survival (DMFS) in ERα (+) breast cancer patients who received tamoxifen. High UCH-L1 expression is also associated with adverse outcomes of the patients with lymph node positive or negative status before tamoxifen treatment (Figure 5C). These findings suggest that high UCH-L1 expression is significantly correlated with poor therapeutic response in malignancy and poor prognosis in patients with breast cancer.

### Targeting of UCH-L1 enhances the sensitivity of ERα (-) breast cancer cells to tamoxifen and fulvestrant

**Figure 6:**
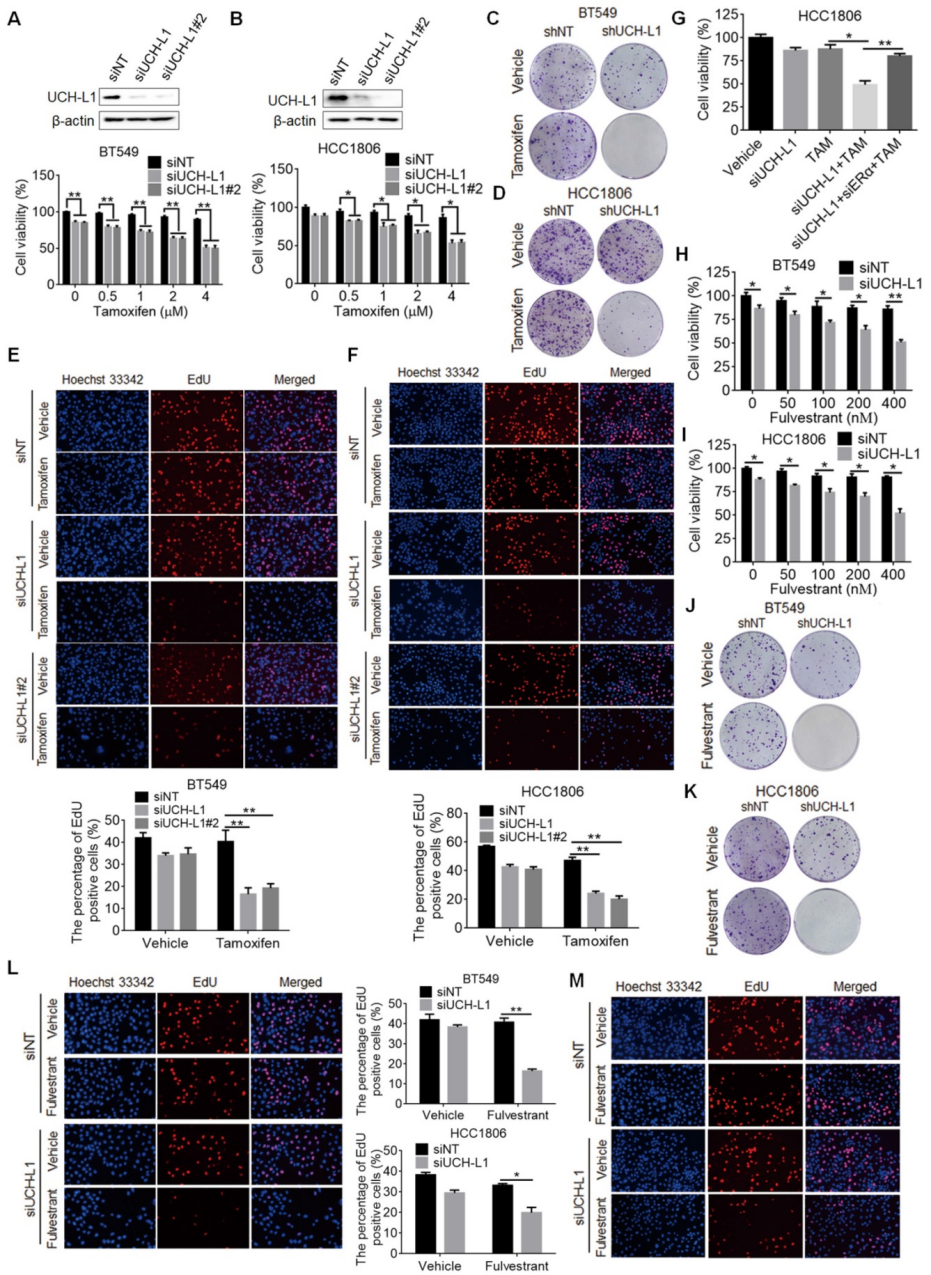
** Inhibition of UCH-L1 increases tamoxifen and fulvestrant sensitivity in ERα (-) cancer cells *in vitro*. (A and B)** BT549 or HCC1806 cells were transfected with a non-targeting siRNA or UCH-L1 siRNAs, followed by treatment with tamoxifen for 72h. Cell viability was measured using CCK-8 assay (Mean ± s.d., n=3 biologically independent experiments. ∗, *p <*0.05; ∗∗, *p <*0.01). **(C, D)** Colony formation of BT549 or HCC1806 cells stably expressing an UCH-L1-targeted shRNA or a control shRNA with 4μM tamoxifen treatment.** (E and F)** BT549 or HCC1806 cells were transfected with a non-targeting siRNA or UCH-L1 siRNAs, followed by treatment with 4μM tamoxifen for 72h. Cells proliferation capacity was detected by EdU. Magnification, ×200.** (G)** HCC1806 cells with UCH-L1 knockdown were transfected with an ERα siRNA, followed by treatment with 4μM tamoxifen for 72h. Cell viability was measured using CCK-8 assay.** (H, I)** BT549 or HCC1806 cells were transfected with a non-targeting siRNA or an UCH-L1 siRNA, followed by treatment with fulvestrant for 72h. Cellular viability was measured using CCK-8 assay.** (J, K)** Colony formation of BT549 or HCC1806 cells stably expressing an UCH-L1-targeted shRNA or a control shRNA and treated with 400nM fulvestrant.** (L, M)** BT549 **(L)** or HCC1806 **(M)** cells were transfected with a non-targeting siRNA or an UCH-L1 siRNA, followed by treatment with 400nM fulvestrant for 72h. Cells proliferation was determined by EdU assay. Magnification, ×200. Results shown are Mean ± s.d., n=3. ∗, *p <*0.05; ∗∗, *p <*0.01.

Finally, we wanted to determine whether targeting UCH-L1 can sensitize ERα (-) cells to tamoxifen. Figure 6A-B show that cytotoxicity of tamoxifen against BT549 and HCC1806 cells was significantly increased when expression of UCH-L1 was silenced, as compared with that in the cells transfected with a non-targeting control RNA. Enhanced cellular sensitivity to tamoxifen was also achieved by co-treatment with LDN (Figure S7A-B). In addition, proliferation of ERα (-) cells with silencing of UCH-L1 expression or with LDN co-treatment was significantly decreased, as compared to the cells treated with tamoxifen alone (Figure 6C-F; Figure S7C-F). Furthermore, in ERα (-) cells we showed that the UCH-L1 knockdown-enhanced sensitivity to tamoxifen was blocked by reduction of ERα (Figure 6G), supporting that the efficacy of tamoxifen caused by UCH-L1 inhibition results from ERα re-expression. To extend these observations, we went on to test whether restoration of ERα expression in ER negative cells through UCH-L1 suppression could sensitize tumor cells to anti-estrogen drug fulvestrant, and observed the similar increased sensitivity to this agent (Figure 6H-M; Figure S7G).

**Figure 7:**
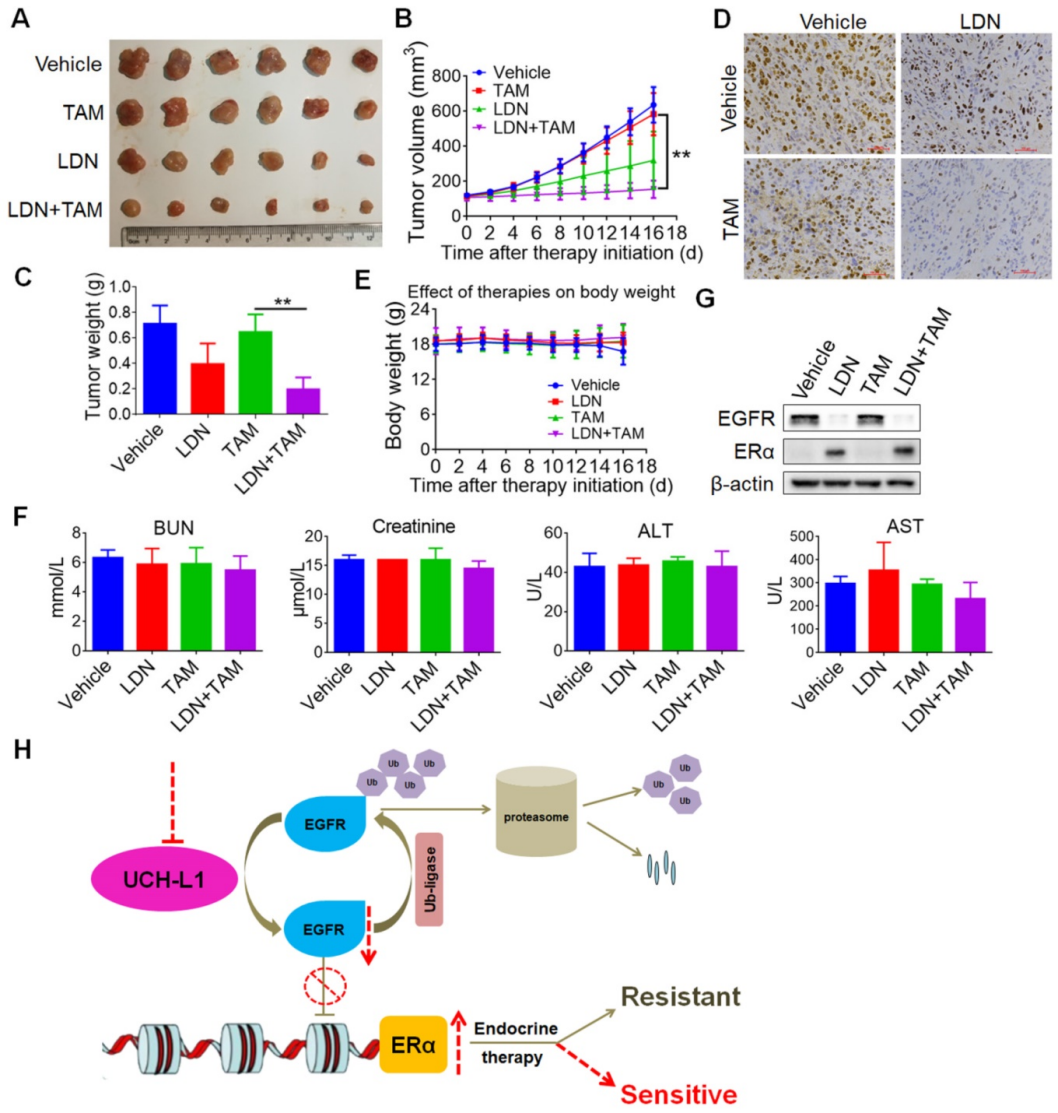
** Inhibition of UCH-L1 increases tamoxifen sensitivity in ERα (-) cancer *in vivo*.** 5-week-old female nude mice were inoculated s.c. with HCC1806 triple negative breast cancer cells. The tumor-bearing mice then received indicated treatment. The tumor sizes were measured on the days as indicated.** (A)** Subcutaneous tumors were excised and photopraphs were taken at the termination of the experiment. **(B)** Tumor sizes were measured on the days as indicated. Data represents the mean ± SD of tumor sizes of each group (n = 6). ∗∗, *P <*0.01.** (C)** Tumor weights were measured at the end of the experiments. Data represents the mean ± SD of tumor weights of each group (n = 6). ∗∗, *P <*0.01.** (D)** Immunohistochemistry staining for Ki67 in the tumor specimens from the mice. **(E)** The effect of treatment on mice body weight. **(F)** Mice liver and kidney functions were measured at the end of the experiments. BUN, blood urea nitrogen; ALT, alanine aminotransferase; AST, aspartate aminotransferase.** (G)** Western blot for EGFR and ERα protein expressions in the xenograft specimens from mice. **(H)** A proposed model for regulation of ERα by UCH-L1. The EGFR protein was maintained dynamic homeostasis by ubiquitin E3 ligases and deubiquitinases orchestrating precisely, while high expression of UCH-L1 broke this balance by de-polyubiquitination, leading to overexpression of EGFR, thereby down-regulating ERα gene transcription. Targeting UCH-L1 could facilitate proteasomal-mediated EGFR degradation, leading to ERα re-expression and re-sensitization to endocrine therapies.

Further, we tested *in vivo* whether inhibition of UCH-L1 could increase effectiveness of tamoxifen in triple negative breast cancer. HCC1806 cells were subcutaneously injected into nude mice, and then the combined treatment of LDN (0.4mg/kg via intraperitoneal injection daily) with tamoxifen (1 mg per dose via oral gavage daily) was given to the tumor-bearing animals. Consistent with our observation *in vitro*, LDN treatment greatly enhanced the efficacy of tamoxifen in this mice xenograft tumor model (Figure 7A-D), without significant changes in body weight (Figure 7E) and cytotoxicity to the liver and kidney (Figure 7F). In addition, the down-regulation of EGFR and up-regulation of ERα proteins were observed in the LDN-treated tumors *in vivo* (Figure 7G). These results imply that therapeutic targeting of UCH-L1 may be further explored as a new approach to restoring sensitivity to anti-estrogen therapy in hormone therapy-insensitive human breast cancer.

## Discussion

Estrogen receptor is a useful predictive marker and a prognostic factor in clinical management of breast cancer. Loss or reduction of ER is the major cause for the insensitivity of breast cancer to hormonal therapy; thus understanding the causes and mechanisms underlying the alterations of ER status may provide novel therapeutic strategy to treatment of ERα-negative breast cancer. There was a study suggesting that high levels of UCH-L1 correlated with negative ERα status and advanced tumor stage 37, yet whether UCH-L1 indeed has a role in the regulation of ERα remains largely unknown. In this study, we demonstrate that UCH-L1 represses ERα expression, and inhibition of UCH-L1 restores ERα expression in ERα (-) breast cancer cells, indicating that UCH-L1 is a negative regulator of ERα. Since the intrinsic or acquired resistance to the selective estrogen receptor modulators is a major obstacle in the management of breast cancers, our studies of UCH-L1 as a regulator of ERα may explain, at least in part, why ERα content is low or lost in a fraction of breast cancers, and may provide a basis for new approaches to up-regulating or maintaining ERα level. Also, we found that HER2+ breast cancer without expression of ERα has low expressions of UCH-L1 (Figure 1C-D; Table S1), indicating that there are different levels of UCH-L1 in the HER2+ and triple negative breast cancer subtypes.

The selective estrogen receptor modulators such as tamoxifen are the most effective and commonly used anti-estrogen agent in treatment of breast cancer. Clinically, about two-thirds of breast cancer are estrogen receptor alpha (ERα) (+) and respond to endocrine therapy such as tamoxifen. However, ERα (-) cancer remains a challenging problem in clinical treatment with hormonal therapy because of the absence of ERα expression. Our study shows that up-regulation of ERα through inhibition of UCH-L1 can render ERα (-) cell sensitivity to tamoxifen treatment. Therefore, UCH-L1 may be a potential therapeutic target for management of patients with ERα (-) breast cancer and respond poorly to hormonal therapy. Combination of tamoxifen with UCH-L1 inhibitor may be worth testing as a new strategy for improving the therapeutic outcome of patients with hormone-resistant breast cancer.

Several studies have demonstrated the inverse relation between ERα and EGFR, and ERα (-) tumors frequently overexpress EGFR that inhibits ERα transcription by activating MAPK 18, 19, 48-50. Previous studies showed that two potential mechanisms may underlie the MAPK-mediated ERα repression. Hyperactivation of MAPK by EGFR overexpression leads to enhanced NF-kB-mediated transcriptional activity, and subsequently decreases ERα expression 51. Consistently, the activity of NF-kB is elevated in ERα (-) breast cancers 52. In addition, hyperactivation of MAPK can stimulate DNMT expression and then link to hypermethylation of the ERα promoter 53. Except for MAPK, PI3K-Akt pathway also participates in the down-regulation of ERα by EGFR. Overexpression of EGFR activates downstream PI3K-Akt signaling pathway, leading to foxo3a phosphorylation and nuclear export and suppression of ERα transcription 54, 55. Thus, mutiple mechanisms may account for the ERα inhibition by EGFR. Up-regulated UCH-L1 could promote the expression level of EGFR, thereby enhancing the invasion and metastasis abilities of tumor cells 47. However, the molecular mechanisms behind remain unknown. Here, we show the evidence that UCH-L1, as a deubiquitinase, promotes the stabilization of EGFR protein by the ubiquitin-proteasome pathway, leading to suppression of ERα transcription. Accumulating studies indicate that the expression of UCH-L1 is closely associated with cancer progression; however, the exact role of UCH-L1 and its regulation in cancer remains incompletely understood. Our results of the regulatory role of UCH-L1 in EGFR stability will help further understand the functions of this deubiquitinating enzyme in cancer development, progression and treatment. UCH-L1 has been found overexpressed in many cancers and considered as a tumor promoting protein. However, the function of UCH-L1 in tumor initiation, progression and invasion is still controversial, as UCHL1 methylation has been reported in multiple tumors, including nasopharyngeal 56, esophageal 57, gastric 58, renal 59, head and neck squamous cell carcinoma 60, hepatocellular 61, and ovarian cancers 62, supporting a critical role in tumor suppression. We found the differential expression of UCH-L1 in ERα (+) and (-) breast cancer cells, suggesting that the potential role of UCH-L1 as an oncogene or a tumor suppressor may be cell context, depending on genetic background or different tissues.

EGFR is a valuable target in cancer treatment 63. Small molecular tyrosine kinase inhibitors (TKIs) such as gefitinib or erlotinib, or monoclonal antibodies such as cetuximab, which target EGFR, have exhibited considerable advantages than conventional chemotherapy. Nevertheless, only patients bearing the special EGFR mutations, including deletion mutations around codons 746-750 in exon 19 and the substitution of leucine with arginine at codon 858 in exon 21, are strong responders to TKIs 64, 65. Moreover, patients who are initially sensitive to TKIs ultimately will develop drug resistance, and this is mainly attributed to the secondary mutation of the EGFR gene 66, 67. In the context of restore expression of ERα, inhibiting UCH-L1 may represent a new therapeutic approach to treatment of malignant breast cancer with loss or reduction of ERα.

In addition, the high expression of UCH-L1 in TNBC cells implies that it may be associated with progression and metastasis of TNBC cells. It has been previously reported that the increased expression of UCH-L1 in TNBC cancer cell lines promoted cell invasion through activating Akt signaling pathway 34. Furthermore, high UCH-L1 expression was shown to be correlated with negative ER, advanced tumor stage and a shorter overall survival of breast cancer patients 37; however, the correlation of UCH-L1 expression with survival of TNBC patients remains undefined. Nevertheless, based on the reported studies including our own, UCH-L1 may be a potential biomarker for predicting the prognosis of breast cancer patients, especially in TNBC.

Taken together, we show that overexpression of UCH-L1 contributes to loss or reduction of ERα in breast cancer, and this is mediated by its deubiquitinating and stabilizing effects on EGFR, which transcriptionally represses the expression of ERα. Targeting UCH-L1 may increase and maintain ERα level by promoting the degradation of EGFR, thereby sensitizing ERα (-) breast cancer cells to the selective ER modulator such as tamoxifen (Figure 7H). The findings reported here not only provide a basis for new approaches to up-regulating ERα level, but also suggest that UCH-L1 may serve as a new adjuvant target for treating human breast cancer with loss or reduction of ERα.

## Supplementary Material

Supplementary figures and table.
