# Supplementary material for: UCH-L1-mediated Down-regulation of Estrogen Receptor α Contributes to Insensitivity to Endocrine Therapy for Breast Cancer
Source: Theranostics. 2020 Jan 1;10(4):1833–48. doi: 10.7150/thno.39814 (PMC6993235; doi:10.7150/thno.39814)
Supplement: Supplementary file 1 — Supplementary figures and table. [file thnov10p1833s1.pdf]

Supplementary Table S1. The results of IHC analysis for breast tumor samples

Intrinsic subtypes	UCHL1		*Row Totals*	Person X^2	p
	Positive	Negative			
Luminal A	2	45	47	32.338	<0.0001***
Luminal B	2	46	48		
HER2 positive	0	45	45		
Triple negative	10	19	29		
Column Totals	14	145	159		

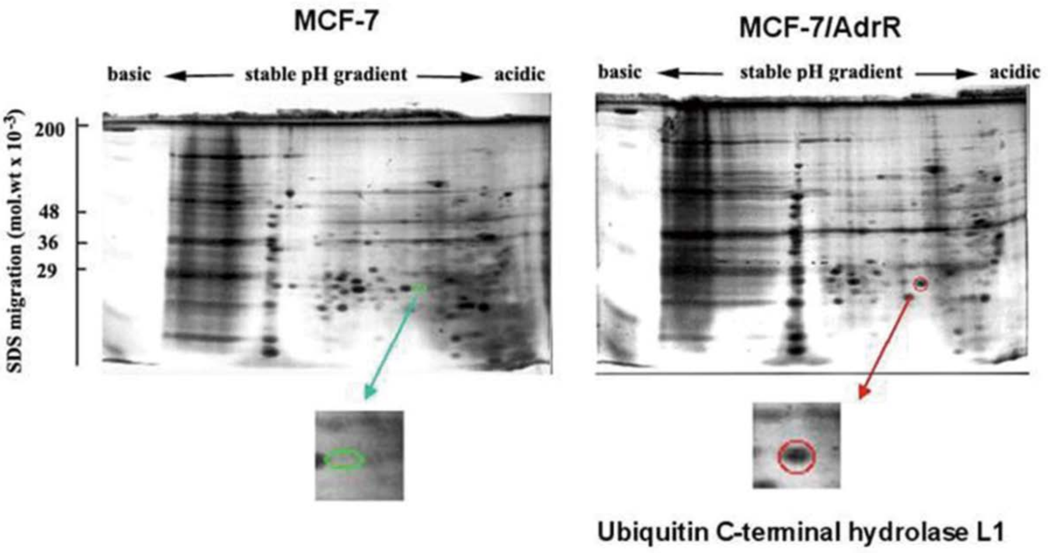

Figure S1. UCH-L1 is overexpressed in MCF-7/AdrR cells. Related to Figure 1. Equal amount of proteins from cell lysates of both MCF-7 and MCF-7/AdrR cell lines were mapped using two-dimensional gel electrophoresis. Proteins were visualized with silver staining. A protein spot with molecular weight of 30 kD and PI of 5.33 was abundant in MCF-7/AdrR cells, but not detectable in MCF-7 cells. The NH2-terminal sequencing of this protein revealed 100% homology with UCH-L1.

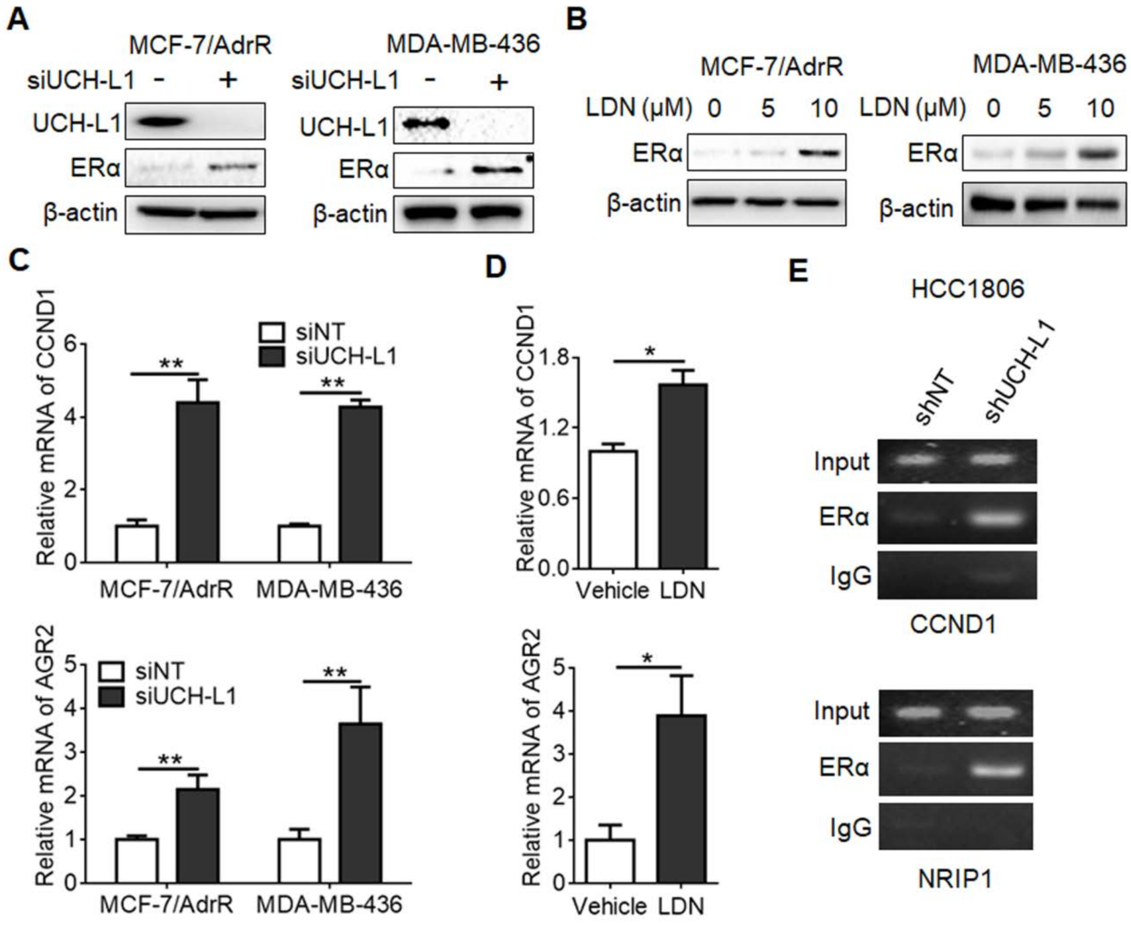

Figure S2. Effects of UCH-L1 siRNA or small molecule inhibitor on ERα in ERα (-) breast cancer cells. Related to Figure 2. MCF-7/AdrR or MDA-MB-436 cells were transfected with a non-targeting siRNA or an UCH-L1 siRNA for 72h (**A**), or were treated with UCH-L1 inhibitor LDN with the indicated concentrations for 24h (**B**). The expressions of UCH-L1 and ERα were measured by western blot. β-actin was used as a loading control. MCF-7/AdrR or MDA-MB-436 cells were transfected with a non-targeting siRNA or an UCH-L1 siRNA for 72h (**C**), MDA-MB-436 cells were treated with 10 μM LDN for 24h (**D**). The mRNA levels of CCND1 and AGR2 were analyzed by real-time PCR (Mean ± s.d., n=3 biologically independent experiments. $*$, p <0.05; $**$, p

<0.01). (**E**) Chromatin immunoprecipitation assay analyzing the binding of ERα to the CCND1/NRIP1 promoter regions in the presence of 10nM E2 for 24 hours. Samples were amplified by PCR and followed by analyzing by agarose gel electrophoresis. Immunoglobulin G (IgG) was used as a negative control.

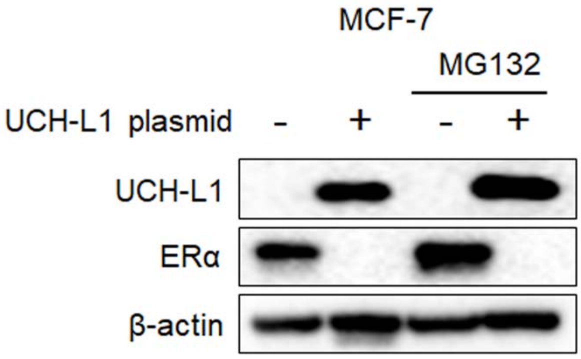

Figure S3. UCH-L1 does not affect the degradation of ERα protein. Related to Figure 3. MCF-7 cells were transfected with a control plasmid or a myc-his-UCH-L1 plasmid, followed by treatment with 20μM MG132 for 4h. The expressions of UCH-L1 and ERα were measured by western blot. β-actin was used as a loading control.

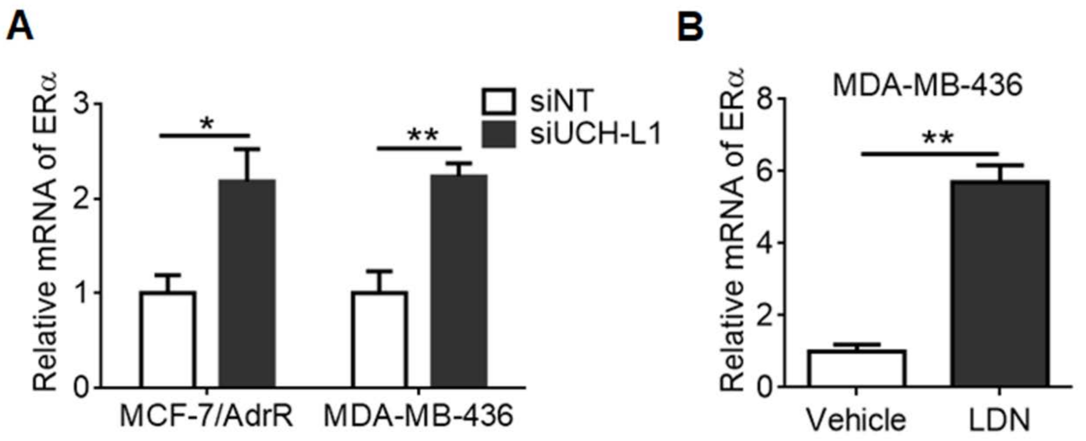

Figure S4. **Effect of UCH-L1 on the transcription of ERα gene.** Related to Figure 3. **(A)** MCF-7/AdrR or MDA-MB-436 cells were transfected with a non-targeting siRNA or an UCH-L1 siRNA for 72h. **(B)** MDA-MB-436 cells were treated with 10 μM LDN for 24h. The ERα mRNA level was analyzed by real-time PCR. Results shown are Mean ± s.d., n=3 biologically independent experiments. ∗, p <0.05; ∗∗, p <0.01.

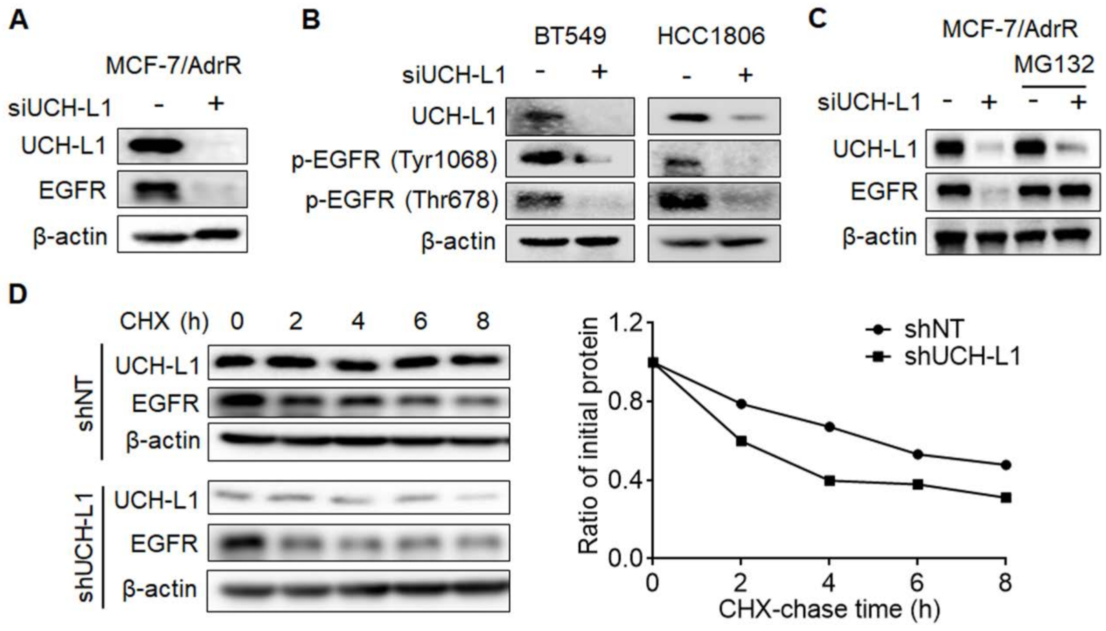

Figure S5. Inhibition of UCH-L1 increases the degradation of EGFR. Related to Figure 4. **(A)** MCF-7/AdrR cells were transfected with a non-targeting siRNA or an UCH-L1 siRNA. The expressions of UCH-L1 and EGFR were measured by western blot. β-actin was used as a loading control. **(B)** BT549 or HCC1806 cells were transfected with a non-targeting siRNA or an UCH-L1 siRNA for 72h. The expressions of UCH-L1 and p-EGFR at Y1068 and Thr678 were examined by western blot. β-actin was used as a loading control. **(C)** MCF-7/AdrR cells were transfected with a non-targeting siRNA or an UCH-L1 siRNA, followed by treatment with 20μM MG132 for 4h. Expressions of UCH-L1 and EGFR were examined by western blot. β-actin was used as a loading control. **(D)** MCF-7/AdrR cells were transfected with a non-targeting shRNA or an UCH-L1 shRNA, and then subjected to cycloheximide (10μg/ml) chase at the indicated time. The expressions of UCH-L1 and

EGFR were measured by western blot. β-actin was used as a loading control.

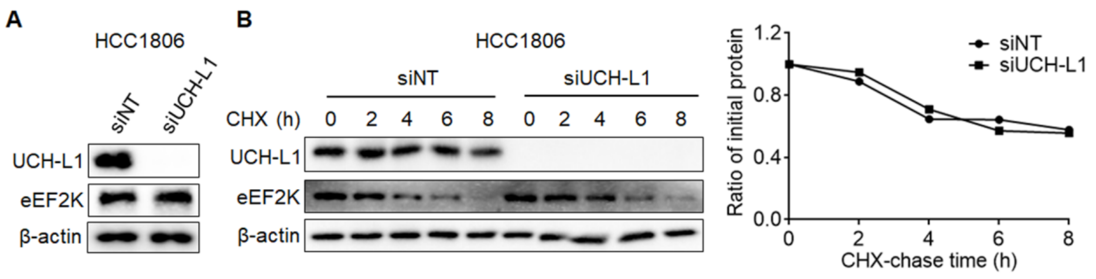

Figure S6. UCH-L1 has no effect on eEF2K protein expression and degradation. Related to Figure 4. **(A)** HCC1806 cells were transfected with a non-targeting siRNA or an UCH-L1 siRNA for 72h. The expressions of UCH-L1 and eEF2K were measured by western blot. β-actin was used as a loading control. **(B)** HCC1806 cells were transfected with a non-targeting siRNA or an UCH-L1 siRNA, and then subjected to cycloheximide (10μg/ml) chase at the indicated time. Expressions of UCH-L1 and eEF2K were measured by western blot. β-actin was used as a loading control.

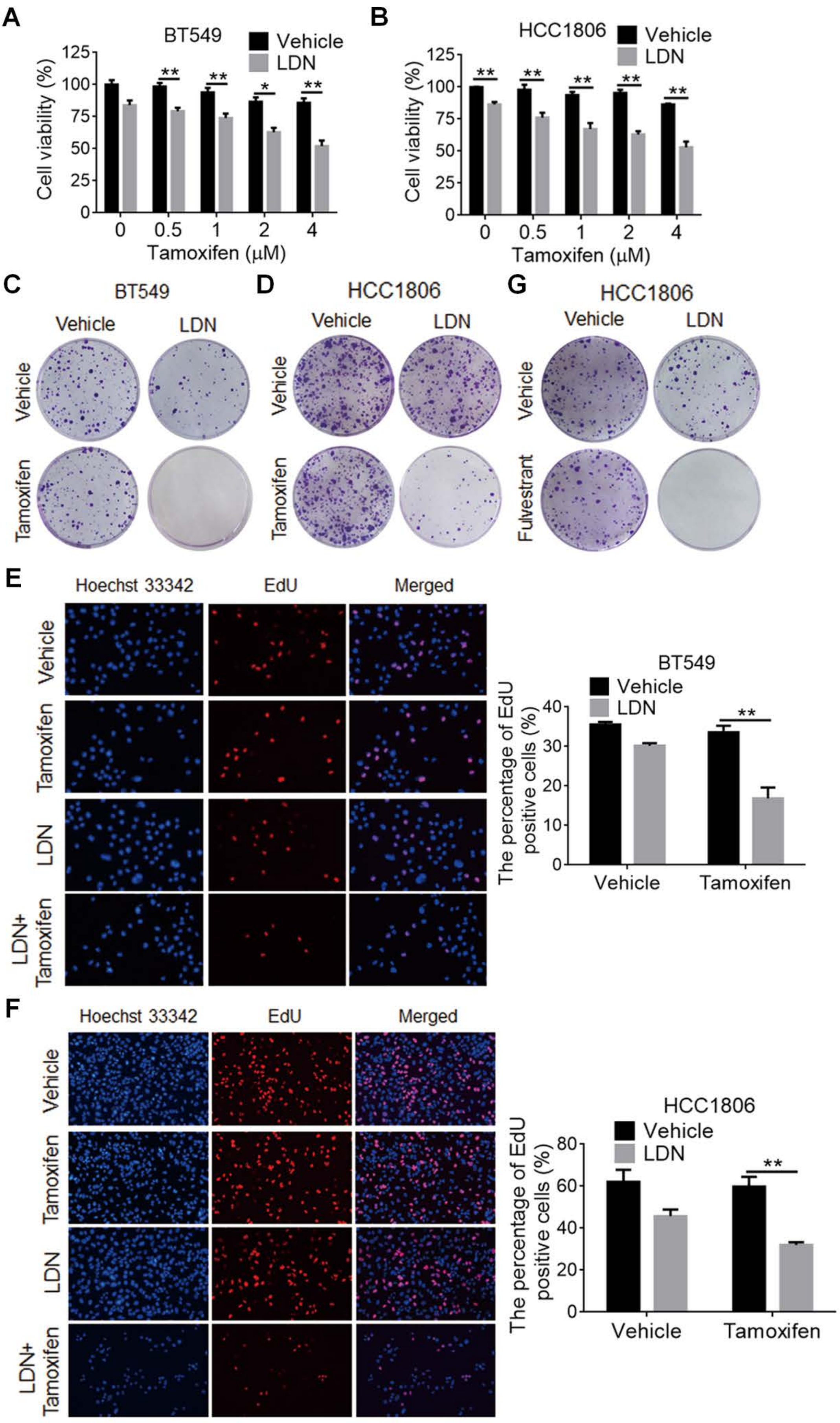
A
BT549
Vehicle
LDN
Cell viability (%)
Tamoxifen (μM)
B
HCC1806
Vehicle
LDN
Cell viability (%)
Tamoxifen (μM)
C
BT549
Vehicle
LDN
Vehicle
Tamoxifen
D
HCC1806
Vehicle
LDN
Vehicle
Tamoxifen
G
HCC1806
Vehicle
LDN
Vehicle
Fulvestrant
E
Hoechst 33342
EdU
Merged
Vehicle
Tamoxifen
LDN
LDN+ Tamoxifen
BT549
Vehicle
LDN
The percentage of EdU positive cells (%)
Vehicle
Tamoxifen
F
Hoechst 33342
EdU
Merged
Vehicle
Tamoxifen
LDN
LDN+ Tamoxifen
HCC1806
Vehicle
LDN
The percentage of EdU positive cells (%)
Vehicle
Tamoxifen

Figure S7. Inhibition of UCH-L1 sensitizes ERα (-) breast cancer cells to tamoxifen and fulvestrant. Related to Figure 6. **(A, B)** BT549 or HCC1806 cells were treated with tamoxifen for 72h in the presence or absence of 10μM LDN. Cell viability was measured using CCK-8 assay. **(C, D)** Colony formation of BT549 or HCC1806 cells after treatment with 4μM tamoxifen in the presence or absence of 10μM LDN. **(E, F)** BT549 or HCC1806 cells were treated with 4μM tamoxifen for 72h in the presence or absence of 10μM LDN. Cells proliferation capacity was detected by EdU. Magnification, ×200. Results shown are Mean ± s.d., n=3. ∗, p <0.05; ∗∗, p <0.01. **(G)** Colony formation of HCC1806 cells after treatment with 400nM fulvestrant in the presence or absence of 10μM LDN.
